# Oral Food Supplement with Bio-Activated Silicium and Vitamins D3 and K2 in the Conservative Management of Osteoporotic Vertebral Compression Fractures

**DOI:** 10.3390/jcm15135206

**Published:** 2026-07-03

**Authors:** Roberto Gazzeri, Marcelo Galarza, Felice Occhigrossi, Christian Carulli, Stefano Telera, Jacopo Mosca, Matteo Luigi Giuseppe Leoni

**Affiliations:** 1 Interventional and Surgical Pain Management Unit, San Giovanni-Addolorata Hospital, 00184 Rome, Italy; 2 Regional Service of Neurosurgery, “Virgen de la Arrixaca” University Hospital, 30001 Murcia, Spain; 3 Orthopaedic Clinic, Careggi University Hospital, University of Florence, 50134 Florence, Italy; 4 Neurosurgery Unit, IRCCS Regina Elena National Cancer Institute, Via Elio Chianesi 53, 00144 Rome, Italy; 5 Anesthesiology, Critical Care and Pain Medicine, La Sapienza University of Rome, 00185 Rome, Italy; 6 Department of Medical and Surgical Sciences and Translational Medicine, Sapienza University of Rome, 00100 Rome, Italy

**Keywords:** osteoporotic vertebral compression fractures, orthosilicic acid, vitamin D3, vitamin K2, fracture healing, conservative management, bone mineralization

## Abstract

**Background**: Osteoporotic vertebral compression fractures (OVCFs) are the most prevalent manifestation of osteoporotic skeletal disease, associated with severe pain, functional decline, and elevated risk of subsequent fractures. Conservative management remains the first-line approach for stable fractures, yet pain control is often suboptimal, and vertebral collapse progresses in up to 37% of patients. Bio-activated orthosilicic acid combined with vitamins D3 and K2 (BioSi-DK) may support fracture healing through complementary mechanisms acting on osteoblast differentiation, collagen synthesis, osteocalcin carboxylation, and mineralization, but its clinical efficacy in OVCFs has not previously been investigated. **Methods**: A retrospective, multi-center comparative cohort study was conducted in patients aged >50 years with DXA-confirmed osteoporosis and acute thoracolumbar OVCFs (AO Spine OF1-OF2) managed conservatively. Patients receiving BioSi-DK supplementation (two capsules daily for two months, then one capsule daily for four months) in addition to standard conservative treatment were compared with controls receiving conservative treatment alone. Propensity score matching (1:1, sex-exact constraint, caliper 0.3 SD) was applied across twelve pre-specified baseline covariates. The primary outcome was pain intensity at six months, assessed by numerical rating scale (NRS). Secondary outcomes included NRS change, analgesic use, Patient Global Impression of Change (PGIC), requirement for vertebral augmentation (kyphoplasty), MRI marrow edema score (MES), and Genant grade change. **Results**: After propensity score matching, 38 patients (19 per group) with balanced baseline characteristics were analyzed (mean age 71.2 ± 6.5 years; 89.5% female; mean T-score −2.61 ± 0.32; mean baseline NRS 8.26 ± 0.95). The BioSi-DK group achieved a significantly lower post-treatment NRS score compared with controls (2.05 ± 2.17 vs. 3.84 ± 2.83; *p* = 0.015; Cohen’s d = −0.71) and a significantly greater mean NRS reduction (−6.21 ± 1.90 vs. −4.42 ± 2.12 points; *p* = 0.005; d = −0.89). Analgesic discontinuation was more frequent in the BioSi-DK group (68.4% vs. 36.8%; *p* = 0.068). Kyphoplasty was required in 5.3% of BioSi-DK patients versus 21.1% of controls (*p* = 0.340; OR = 0.21), and vertebral compression grade remained stable in 100% of supplemented patients versus 84% of controls. At two months, MES improvement by at least one category was more frequently observed in the BioSi-DK group than in controls, suggesting an earlier edema resolution effect; at six months, MES distribution was comparable between groups (*p* = 0.620). **Conclusions**: BioSi-DK supplementation as an adjunct to conservative management was associated with a statistically significant and clinically large reduction in pain at six months, with favorable trends in analgesic burden, kyphoplasty requirement, and edema resolution. The safety profile was excellent. These findings support the conduct of prospective, randomized, placebo-controlled trials to confirm BioSi-DK as an effective adjunct therapy for OVCFs.

## 1. Introduction

Osteoporotic vertebral compression fractures (OVCFs) are the most common manifestation of osteoporotic skeletal disease and represent a major global public health burden [1]. Osteoporosis affects approximately 200 million individuals worldwide, with OVCF incidence rates of 10.7 per 1000 person-years in women and 5.7 per 1000 person-years in men over the age of 50 [2]. OVCFs are associated with significant reductions in health-related quality of life, progressive spinal deformity, and increased mortality risk [3,4]. Moreover, OVCFs substantially increase the risk of subsequent vertebral fractures by up to fivefold, initiating a cascade of skeletal fragility that further worsens long-term prognosis [5,6].

Most OVCFs are initially managed conservatively, and current international guidelines recommend non-operative treatment for stable fractures without neurological compromise [7,8,9]. Conservative management typically includes relative bed rest, thoracolumbar orthosis, multimodal analgesia, and early mobilization. Although pain usually improves within 6–12 weeks, vertebral collapse progresses in 7–37% of cases, sometimes requiring surgical intervention [10]. Analgesic strategies frequently include non-steroidal anti-inflammatory drugs (NSAIDs), opioids, and adjuvant agents [11]. When conservative management fails, vertebral augmentation procedures such as vertebroplasty and balloon kyphoplasty may be performed to achieve rapid pain relief through mechanical stabilization of the fractured vertebral body [12,13]. While effective for short-term analgesia, these procedures carry risks including cement leakage and adjacent-level fractures, which occur in 2.5–17.3% of patients [14,15].

These limitations have prompted increasing interest in therapeutic strategies that support bone healing and improve outcomes during the conservative management phase. Magnetic resonance imaging (MRI) is the reference modality for evaluating OVCFs, particularly through short tau inversion recovery (STIR) sequences that detect bone marrow edema (BME) and distinguish acute from chronic fractures [16,17]. The extent of vertebral BME has prognostic significance, with more extensive edema associated with delayed healing and a higher risk of vertebral collapse [18].

Pharmacological management of osteoporosis is an essential component of OVCF treatment. Anti-resorptive therapies such as bisphosphonates and selective estrogen receptor modulators reduce fracture risk, while anabolic agents such as teriparatide may improve bone density and reduce pain [3,19]. However, these treatments may be limited by cost, side effects, and adherence issues, highlighting the need for additional well-tolerated adjunctive strategies [20,21]. Vitamin D3 plays a central role in bone metabolism by enhancing calcium absorption and regulating osteoblast and osteoclast activity through the RANK/RANKL/OPG pathway [22,23]. Vitamin D deficiency is common in elderly osteoporotic populations and is associated with impaired bone mineralization and delayed fracture healing [24]. Vitamin K2 (menaquinone-7) contributes to bone health by enabling gamma-carboxylation of osteocalcin, a process essential for effective calcium binding and mineralization of the bone matrix [25]. Silicon, particularly in the form of orthosilicic acid, has also emerged as an important trace element in bone biology, promoting collagen synthesis, osteoblast differentiation, and inhibition of osteoclastogenesis [26].

The complementary biological actions of bio-activated orthosilicic acid, vitamin D3, and vitamin K2 suggest a potential synergistic role in supporting fracture healing and bone mineralization. It should be noted, however, that the broader literature on individual micronutrient supplementation for fracture healing is not uniformly positive: while some trials of vitamin D, vitamin K2, or silicon have reported improvements in bone mineral density or bone turnover markers, others, particularly large vitamin D supplementation trials, have shown neutral effects on fracture incidence and high-quality evidence for orthosilicic acid in clinical fracture healing remains limited [27,28,29,30,31]. The present combined formulation is therefore best regarded as a biologically rationalized adjunct whose clinical value has yet to be established. However, the clinical efficacy of this combined supplementation strategy has not been investigated in patients with OVCF managed conservatively. The aim of the present study was therefore to evaluate the clinical and radiological efficacy of an oral dietary supplement combining bio-activated orthosilicic acid with vitamins D3 and K2 (BioSi-DK) as an adjunct to standard conservative treatment in patients with acute thoracolumbar OVCFs. Particular attention was given to pain reduction, fracture healing assessed by MRI BME scoring, analgesic requirements, and the rate of conservative treatment failure requiring vertebral augmentation.

## 2. Methods

This was a retrospective comparative cohort study. Between January 2019 and September 2024, consecutive patients diagnosed with thoraco-lumbar OVCFs, classified as AO Spine type OF1 or OF2 [32], who underwent conservative management were identified from the institutional registry and screened for eligibility. The study was conducted in accordance with the ethical standards of the institutional research committee and the principles of the 1964 Declaration of Helsinki and its later amendments (IRB approval number: 30370_spe). Informed consent was obtained from all participants.

Patients were included if they met all of the following criteria: were older than 50 years at the time of fracture; had acute onset of back pain following minor trauma within the preceding 8 weeks, or a spontaneous vertebral fracture without an identifiable traumatic event; and had dual-energy X-ray absorptiometry (DXA)-confirmed osteoporosis, defined as a lumbar spine (L2–L4) T-score of −2.0 standard deviations or lower and availability of both baseline and six-month follow-up thoracolumbar MRI examinations. Patients were excluded if the vertebral fracture was secondary to infection, primary bone tumors (such as hemangioma or multiple myeloma), or metastatic disease; if they had previously undergone vertebroplasty, balloon kyphoplasty, or posterior spinal fixation at the affected vertebral level; or if medical records were incomplete or the MR imaging of the thoracic and lumbar spine was non-diagnostic.

### 2.1. Treatment Protocol

All patients received a standardized conservative treatment protocol comprising an initial period of bed rest (one to two weeks, titrated to pain intensity), thoracolumbar orthosis bracing, and analgesic therapy (e.g., tramadol and paracetamol) administered according to the WHO analgesic ladder, together with anti-osteoporotic treatment prescribed by endocrinologists in accordance with current clinical guidelines [33,34]. Patients were allocated to one of two groups based on a clinician’s decision, and no randomization was performed prior to the group assessment. Treatment assignment was therefore non-randomized and reflected the prescribing physician’s clinical judgment and local practice rather than pre-defined protocolised criteria, with BioSi-DK offered as an optional adjunct during the study period. All patients in both groups received the same standard anti-osteoporotic pharmacotherapy, consisting of oral bisphosphonates combined with vitamin D supplementation, and no patient received denosumab, teriparatide, or other anabolic agents. Because this background therapy was uniform across the two study arms, it could not act as a differential confounder of the between-group comparison. The control group received conservative treatment alone, whereas the treated group received the same conservative regimen supplemented with the oral dietary formulation BioSi-DK (bio-activated orthosilicic acid with vitamins D3 and K2; Sildì, Geopharma, Bari, Italy). The supplement was administered as two soft-gel capsules daily for the first two months, followed by one soft-gel capsule daily for the subsequent four months (total supplementation duration: six months). Each soft-gel capsule provided bio-activated orthosilicic acid in highly bioavailable monomeric form, obtained through a patented manufacturing process (Mesoporosil; patent WO2019179611A1), together with vitamin D3 (cholecalciferol) 30 µg (1200 IU; 600% of the EU nutrient reference value) and vitamin K2 (menaquinone-7) 45 µg (60% of the EU nutrient reference value). The daily intake during the initial two-month phase, therefore, corresponded to 2400 IU vitamin D3 and 90 µg menaquinone-7, decreasing to 1200 IU vitamin D3 and 45 µg menaquinone-7 for the subsequent four months. BioSi-DK is intended to support bone matrix mineralization and fracture healing through the action of bio-activated silicon, which stimulates osteoblast activity and type I collagen synthesis, together with vitamins D3 and K2, which contribute to calcium absorption and osteocalcin carboxylation, respectively. Adherence to BioSi-DK supplementation, brace use, prescribed analgesics, and anti-osteoporotic medication was verified at each scheduled follow-up visit through direct patient interview and review of the clinical record; all patients included in the present analysis confirmed completion of the prescribed treatment plan, and none were excluded for non-adherence. Balloon kyphoplasty was indicated in cases of resistance to conservative treatment, persistent or progressive pain despite conservative treatments.

### 2.2. Data Collection and Baseline Assessment

Hospital records were reviewed using standardized data collection forms. Baseline variables recorded included: age, sex, body mass index (BMI), smoking status, duration of symptoms prior to presentation, pre-existing comorbidities (diabetes mellitus, chronic obstructive pulmonary disease [COPD], cardiovascular disease, cerebrovascular disease, hypertension), use of analgesic medications, anti-osteoporotic pharmacotherapy, fracture level, mechanism of injury (minor trauma versus spontaneous) and fracture morphology on imaging. Bone mineral density was quantified using DXA of the lumbar spine (L2–L4), with results expressed as T-scores according to WHO diagnostic criteria. All patients underwent weight-bearing anteroposterior and lateral thoracolumbar radiographs at baseline, followed by MRI of the thoracic and lumbar spine performed at baseline, at two-month follow-up, and at six-month follow-up, using a standardized protocol comprising sagittal and axial T1-weighted, T2-weighted, and short tau inversion recovery (STIR) sequences. MRI was used to confirm the symptomatic fracture level and to differentiate acute fractures, characterized by BME on STIR sequences, from chronic or incidental vertebral fractures. BME pattern on STIR sequences was classified into four types using a marrow edema score (MES): type 1, no identifiable marrow edema or edema confined to the fracture line; type 2, mild edema involving less than 25% of the vertebral body; type 3, moderate edema involving 25–75% of the vertebral body; and type 4, severe edema involving 75–100% of the vertebral body [35]. Vertebral compression severity was graded using the Genant semi-quantitative scale [36]: Grade 1, vertebral height reduction of less than 25%; Grade 2, height reduction of 25–50%; and Grade 3, height reduction exceeding 50% relative to the adjacent vertebrae. All MRI examinations were independently reviewed by two board-certified neuroradiologists with more than ten years of experience in musculoskeletal imaging, who were blinded to treatment allocation and to the clinical course of the patients. The BME score and the Genant grade were assigned independently by each reader at every time point and inter-observer reproducibility was quantified using the weighted Cohen’s kappa coefficient. Agreement between readers was excellent for both measures (weighted κ = 0.88 for the BME score and weighted κ = 0.90 for the Genant grade). The few discrepancies were resolved by consensus and the consensus reading was used for all analyses.

### 2.3. Clinical Outcome Measures

Clinical assessments were performed at three time points: baseline (at the time of diagnosis and initiation of treatment), two-month follow-up, and six-month follow-up. Pain intensity was the primary outcome, assessed using the numerical rating scale (NRS), an 11-point patient-reported scale ranging from 0 (no pain) to 10 (worst imaginable pain). The primary efficacy endpoint was the NRS score at six-month follow-up. Patient Global Impression of Change (PGIC) was assessed at six months using the validated seven-point scale [37], ranging from 0, ‘very much worse’, to 7, ‘very much improved’. For binary analyses, patients rating their condition as ‘much improved’ or ‘very much improved’ were classified as responders (PGIC-improved). Analgesic use was categorized at both time points as: ‘No analgesic use’, ‘Occasional use (as needed)’, or ‘Regular use (scheduled)’. A reduction in analgesic category from baseline to follow-up was considered a secondary clinical improvement endpoint. Other endpoints included: requirement for vertebral augmentation considered as a surrogate for conservative treatment failure; duration of additional brace-wearing beyond the initial protocol; MES change from baseline to two months and from two months to six months; Genant grade change at two-month and six-month follow-up; duration of additional brace-wearing beyond the initial protocol; MES change from baseline to six months and Genant grade change from baseline to six months.

**Figure 1:**
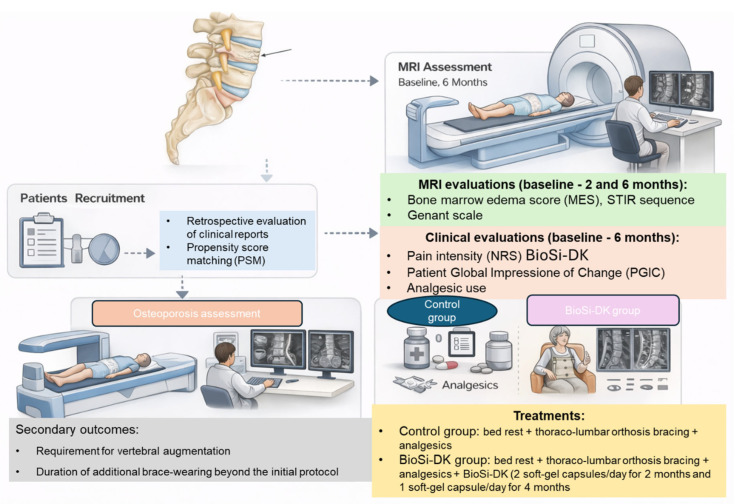
Schematic overview of the study design, treatment protocol, and outcome assessments. Clinical and radiological evaluations were performed at three time points: baseline, two-month follow-up (MRI marrow edema score and Genant grade), and six-month follow-up (all endpoints).

Treatment protocol, clinical and radiological outcomes are summarized in Figure 1.

### 2.4. Statistical Analysis

Descriptive statistics are reported as mean (standard deviation [SD]) for continuous variables and as count (percentage) for categorical variables. Given the non-randomized, retrospective design of this study, propensity score matching (PSM) was employed as the primary analytical strategy to reduce measured confounding and approximate the balance expected from randomization [38]. Propensity scores representing the conditional probability of BioSi-DK assignment given baseline covariates were estimated using multivariable logistic regression incorporating the following twelve pre-specified covariates: age, sex, BMI, lumbar T-score, pre-treatment NRS, pain duration, minor trauma mechanism, and comorbidities (diabetes mellitus, COPD, cardiovascular disease, hypertension, and smoking status). Model discrimination was assessed by the C-statistic (area under the ROC curve). An improved 1:1 nearest-neighbor matching algorithm was applied on the logit of the propensity score with a caliper width of 0.3 standard deviations of the logit-propensity score [39]. To prevent artifactual imbalance in sex, a key determinant of OVCFs, sex-exact matching was imposed as a primary constraint, with a hierarchical fallback protocol: sex-exact matching within caliper; sex-exact matching without caliper; unconstrained nearest-neighbor matching as a last resort to maximize retention of treated patients. This strategy retained all 19 BioSi-DK patients in the matched analysis. Covariate balance before and after matching was quantified using the absolute standardized mean difference (SMD), where an SMD below 0.10 was considered indicative of good balance and an SMD below 0.20 was considered acceptable [40]. All primary and secondary outcome analyses were conducted in the PSM-matched cohort. Continuous outcomes (NRS post-treatment, NRS change, additional brace weeks, MES change, Genant grade change) were compared between groups using the Mann–Whitney U test, given the non-normal distribution of residuals (confirmed by the Shapiro–Wilk test). Effect sizes for continuous outcomes were expressed as Cohen’s d with 95% confidence intervals estimated by bootstrap resampling (1000 iterations). Effect sizes of 0.2, 0.5, and 0.8 were interpreted as small, medium, and large, respectively. Binary outcomes (PGIC-improved, vertebral augmentation requirement) were compared using Fisher’s exact test, with effect sizes expressed as odds ratios (OR) with 95% mid-p exact confidence intervals. Analgesic use was analyzed using the asymptotic Mann–Whitney test with analgesic category coded as an ordinal integer score. The six-month NRS pain score was the single pre-specified primary endpoint; all other measures (NRS change from baseline, analgesic use, PGIC, kyphoplasty requirement, MES change, and Genant grade change) were pre-specified secondary endpoints analyzed in an exploratory manner. Because these secondary analyses were not adjusted for multiple comparisons, the associated *p*-values are descriptive, and the corresponding findings are regarded as hypothesis-generating rather than confirmatory; this approach was adopted to control the family-wise type I error for the primary endpoint while limiting over-interpretation of the secondary endpoints. Given the modest matched sample (19 patients per group), a sensitivity power evaluation indicated approximately 80% power (two-sided α = 0.05) to detect a large between-group difference (Cohen’s d 0.93) in the primary NRS outcome, but substantially lower power for the binary secondary endpoints (kyphoplasty requirement, PGIC responder status and Genant progression); the study was therefore underpowered for these endpoints and their non-significant results should be interpreted as inconclusive rather than as evidence of absence of effect. A two-sided *p*-value of less than 0.05 was considered statistically significant. All analyses were performed using R v4.5.2 (R Foundation for Statistical Computing, Vienna, Austria; www.r-project.org).

## 3. Results

**Figure 2:**
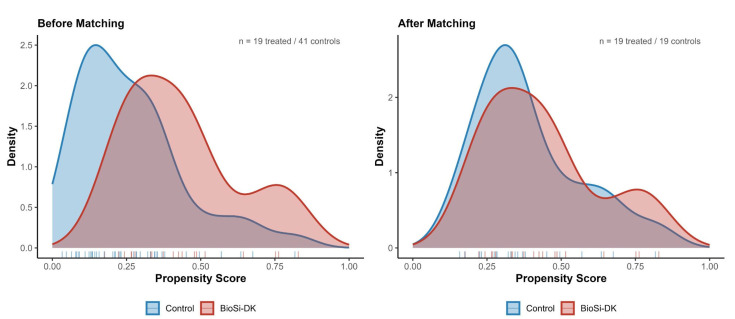
Propensity score overlap density plots before (**left panel**, n = 19 treated vs. 41 controls) and after matching (**right panel**, n = 19 per group). Rug plots indicate individual propensity scores. The substantial overlap in both panels confirms adequate common support and absence of positivity violations.

A total of 60 patients with thoraco-lumbar OVCFs who underwent conservative management were included in the analysis. Of these, 41 patients (68.3%) received standard conservative treatment (control group), while 19 (31.7%) additionally received the BioSi-DK oral silicon-based food supplement (BioSi-DK group). The overall cohort had a mean age of 71.5 ± 6.9 years, was predominantly female (88.3%), and had a mean BMI of 22.7 ± 1.6 kg/m^2^. The median T-score was −2.64 ± 0.34, consistent with established osteoporosis. Fractures were most frequently located at the thoracolumbar junction and lumbar spine, with T12 representing the most commonly affected vertebra (20.6%), followed by L1 (16.2%) and L4 (16.2%), and L3 (14.7%). Lower frequencies were observed at T11 (8.8%), L5 (8.8%), and L2 (7.4%), while upper thoracic levels were rarely involved. Multiple-level fractures (two or more contiguous segments) were present in 13 patients (21.7%). Some imbalance in covariates was evident before propensity score matching, for example, in pain duration before treatment (weeks, mean ± SD: 4.9 ± 2.0 in the control group vs. 5.8 ± 1.4 in the BioSi-DK group; *p* = 0.080). Therefore, to balance covariates and to address the non-randomized treatment allocation and potential confounding, PSM was performed. The distribution of propensity scores before and after matching is illustrated in Figure 2. Prior to matching, the treated and control groups showed partial overlap with differences in distribution; however, after propensity score matching, the density curves demonstrated substantial overlap between groups, indicating adequate common support and absence of positivity violations.

After PSM, a total of 38 patients were included in the final matched cohort, with 19 patients in the BioSi-DK group and 19 in the control group. Baseline demographic and clinical characteristics were well balanced between the two groups (Table 1). No statistically significant differences were observed in age (71.63 ± 7.28 vs. 70.68 ± 5.73 years, *p* = 0.658), BMI (22.87 ± 1.30 vs. 22.87 ± 1.44 kg/m^2^, *p* = 1.000), or baseline T-score (−2.59 ± 0.36 vs. −2.62 ± 0.28, *p* = 0.841). Similarly, baseline NRS was identical in the two groups (8.26 ± 0.99 vs. 8.26 ± 0.93, *p* = 1.000), and the mean pain duration before treatment initiation was comparable (5.47 ± 2.32 vs. 5.79 ± 1.44 weeks, *p* = 0.617). The prevalence of minor trauma preceding the OVCF was identical between groups (36.8% in both groups). Comorbidities were also evenly distributed, including diabetes (26.3% vs. 21.1%), chronic obstructive pulmonary disease (10.5% vs. 15.8%), cardiovascular disease (10.5% vs. 10.5%), hypertension (31.6% vs. 31.6%), and smoking status (42.1% vs. 47.4%), with no significant differences between groups (Table 1).

**Figure 3:**
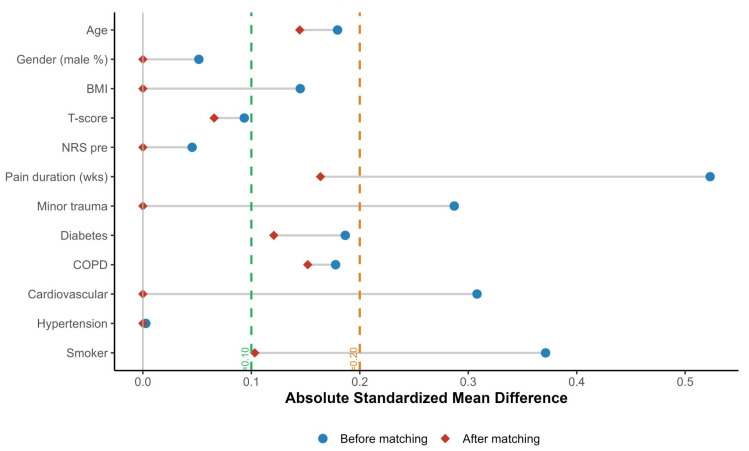
Love plot depicting absolute SMD for each covariate before (blue circles) and after (red diamonds) propensity score matching. The green and orange dashed lines indicate the thresholds of SMD = 0.10 (good balance) and SMD = 0.20 (acceptable balance), respectively. After matching, all covariates fell below the SMD = 0.20 threshold (mean SMD = 0.063, max SMD = 0.164).

SMD for all variables was below 0.20, confirming adequate covariate balance after PSM. The mean SMD decreased from 0.222 in the unmatched cohort to 0.063 after matching, while the maximum SMD was reduced from 0.523 to 0.164. No variable exceeded the pre-specified SMD threshold of 0.20 (Figure 3). The complete baseline characteristics of the full unmatched cohort (n = 60), together with the standardized mean differences before and after matching for every covariate, are provided in Supplementary Table S1.

**Figure 4:**
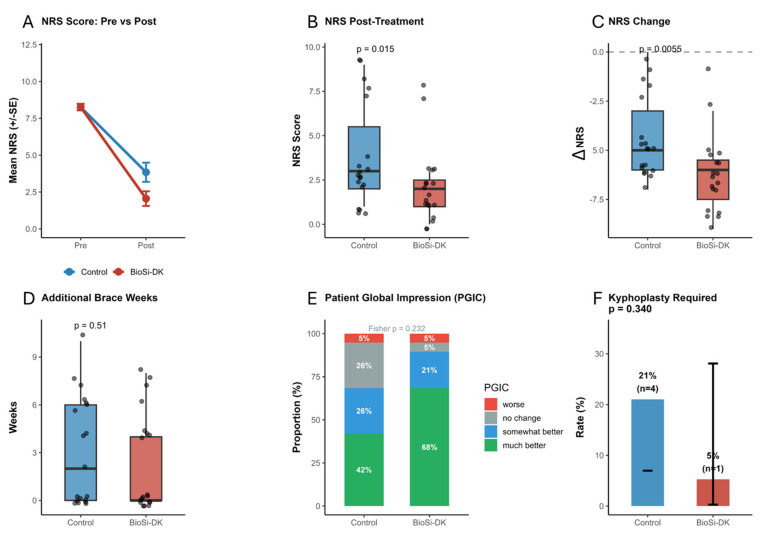
Six-panel summary of clinical outcomes in the matched cohort. (**A**) NRS pain trajectory (pre vs. post, mean ± SE); (**B**) NRS post-treatment boxplot (*p* = 0.015); (**C**) NRS change from baseline (*p* = 0.005). (**D**) additional brace weeks (*p* = 0.51); (**E**) PGIC stacked bar chart (Fisher *p* = 0.232); (**F**) kyphoplasty requirement rate with 95% Wilson confidence intervals (*p* = 0.340). Jittered individual data points are overlaid on boxplots. The delta symbol (∆) in the NRS change panel (**C**) denotes post minus pre values.

In the PSM-matched cohort, BioSi-DK treatment was associated with a significantly greater reduction in pain compared to standard conservative treatment (Figure 4A). The mean post-treatment NRS score was 2.05 ± 2.17 in the BioSi-DK group versus 3.84 ± 2.83 in controls (Mann–Whitney U, *p* = 0.015; Cohen’s d = −0.71, 95% CI [−1.37, −0.05]), indicating a large and clinically meaningful treatment effect (Figure 4B). The mean NRS reduction from baseline was similarly superior in the BioSi-DK group: −6.21 ± 1.90 versus −4.42 ± 2.12 points (*p* = 0.005; Cohen’s d = −0.89, 95% CI [−1.56, −0.22]), representing a difference of approximately 1.8 NRS points in favor of BioSi-DK (Figure 4C). Additional brace wear was similar between groups (2.37 ± 3.08 weeks BioSi-DK vs. 3.11 ± 3.41 weeks control; *p* = 0.506; d = −0.23) (Figure 4D). A clinically meaningful improvement in PGIC (rated as ‘somewhat better’ or ‘much better’) was achieved by 89% of BioSi-DK patients versus 68% of controls (Fisher’s exact, *p* = 0.232) (responder rate 89.5%, 95% CI 68.6–97.1% in the BioSi-DK group vs. 68.4%, 95% CI 46.0–84.6% in controls; OR 3.77, 95% CI 0.65–21.8), (Figure 4E). Although this 21 percentage-point absolute difference did not reach statistical significance, attributable in part to the limited sample size, it represents a clinically relevant trend and an odds ratio of 3.77 in favor of BioSi-DK. Treatment failure necessitating kyphoplasty occurred in 1 of 19 BioSi-DK patients (5.3%) compared with 4 of 19 controls (21.1%) (Fisher’s exact, *p* = 0.340; OR = 0.21, 95% CI [0.02, 1.97]) (Figure 4F).

Post-treatment analgesic use showed a favorable trend in the BioSi-DK group: 68.4% discontinued analgesics entirely (no analgesic use post-treatment) compared with 36.8% of controls. (discontinuation rate 95% CI 46.0–84.6% in the BioSi-DK group vs. 19.1–59.0% in controls; absolute difference 31.6 percentage points, 95% CI −0.5 to 58.0). Only 10.5% of BioSi-DK patients required regular analgesics post-treatment versus 21.1% of controls. Ordinal Mann–Whitney analysis of analgesic use (coded No = 1, Sometimes = 2, Regularly = 3) yielded W = 237.5, Z = 1.84, *p* = 0.068, indicating a borderline-significant trend towards lower post-treatment analgesic burden in the BioSi-DK group.

**Figure 5:**
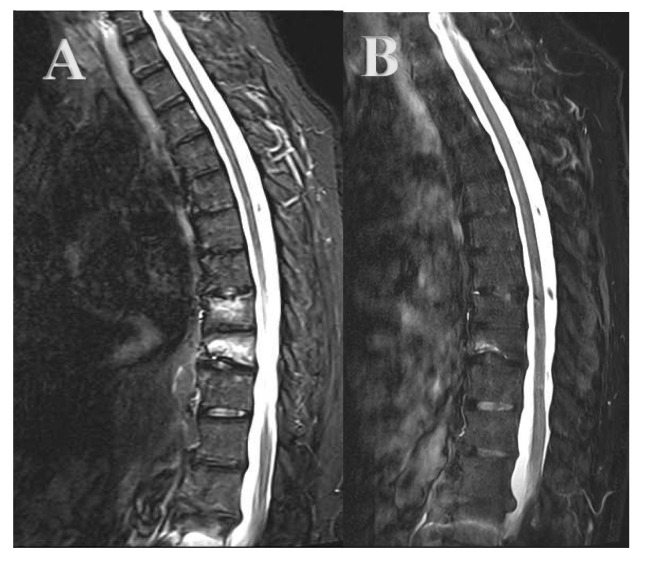
76 y.o. patient with T8 and T9 vertebral body fracture. (**A**) Sagittal short tau inversion recovery (STIR) MR images showing MES 4 at the T9 level and MES 3 at the T8 level. (**B**) STIR MR images after 2 months of conservative treatment and oral food supplement treatment, showing MES 1 at T8 and T9 vertebral bodies.

In the BioSi-DK group, the distribution of MES grades showed a significant shift toward improvement at 2 months, with Grade 1 increasing to 15.8% and Grades 2–3 accounting for 84.2% (*p* = 0.003). This trend continued at 6 months, where Grade 1 increased further to 57.9%, while Grade 3 decreased to 10.5% (*p* = 0.017 vs. 2 months), indicating a progressive improvement over time. In contrast, the control group did not show a statistically significant change at 2 months compared to baseline (*p* = 0.06), with MES distribution of 36.8% Grade 1, 52.6% Grade 2, and 10.5% higher-grade lesions; however, a marked and statistically significant improvement was observed between 2 and 6 months (*p* < 0.001), with Grade 1 increasing to 68.4% and Grade 2 decreasing to 21.1%, while higher-grade lesions remained stable (10.5%) (Figure 5).

Notably, both groups exhibited a marked shift toward lower MES categories between baseline and six months, with no patient experiencing worsening (0% worsening rate). At two months, improvement by at least one MES category was more frequently observed in the BioSi-DK group compared to the control group, supporting an earlier treatment effect. At six months, the post-treatment MES distribution was comparable between groups, with a median score of 1 [IQR 1–2], and no significant between-group difference was detected (Wilcoxon rank-sum W = 165.5, *p* = 0.620; rank-biserial r = 0.08), indicating a negligible effect size and suggesting a similar overall trajectory of MES resolution consistent with the natural healing process of OVCFs.

Vertebral compression severity at baseline was balanced between groups (control: G1 n = 10, G2 n = 9; BioSi-DK: G1 n = 9, G2 n = 10; no G3 fractures in either group). At two-month follow-up, no statistically significant differences were observed between the control and BioSi-DK groups in terms of Genant grade distribution (Wilcoxon rank-sum W = 178.5, *p* = 0.742; rank-biserial r = 0.05), confirming comparable early structural evolution. At six-month follow-up, the BioSi-DK group demonstrated complete grade stability, with no patient experiencing progression to a higher Genant grade (worsening rate 0%; grade-stability rate 100%, 95% CI 83.2–100%). In contrast, three control patients (16%; grade-stability rate 84.2%, 95% CI 62.4–94.5%) showed worsening of vertebral compression grade, including one who progressed to G3. Despite this clinically relevant difference in worsening rates, the between-group comparison of post-treatment Genant grade distributions did not reach statistical significance (Wilcoxon W = 185.0, *p* = 0.894; rank-biserial r = −0.03, negligible effect). Fisher’s exact test comparing grade stability versus any progression likewise did not show a significant difference (*p* = 0.487), likely reflecting limited statistical power given the low overall rate of fracture progression. No adverse events or side effects attributable to the BioSi-DK oral supplement were reported in any patient.

## 4. Discussion

This retrospective, multi-center comparative cohort study evaluated the clinical and radiological efficacy of an oral dietary supplement combining bio-activated orthosilicic acid with vitamins D3 and K2 as an adjunct to standard conservative management in patients with OVCFs. The principal findings of this study indicate that supplementation with BioSi-DK, in addition to standard conservative treatment, was associated with significantly greater pain reduction at six months compared with conservative management alone. A higher proportion of patients in the BioSi-DK group discontinued analgesic therapy, although this difference did not reach statistical significance. Similarly, the proportion of patients requiring balloon kyphoplasty and the rate of PGIC responders were lower and higher in the BioSi-DK group, respectively, but these differences were not statistically significant. Radiological outcomes, including changes in the marrow edema score, were comparable between groups without significant differences, while vertebral compression stability showed a favorable but non-significant trend in supplemented patients. No treatment-related adverse events were observed during the study period.

OVCFs represent the most prevalent manifestation of osteoporotic skeletal disease, and their management remains a subject of ongoing international debate [2]. Current evidence-based guidelines, including those of the German Society for Orthopaedics and Trauma (DGOU) and the North American Spine Society (NASS), endorse conservative treatment as the primary strategy for stable fractures without neurological compromise, particularly for AO Spine OF1 and OF2 morphologies [7,8,9].

The study by Lee et al. [41], enrolling 259 patients with OVCFs in a prospective comparative design, found that approximately 65% of patients could be managed successfully without surgical escalation, and identified older age, severe osteoporosis (T-score below −2.95), overweight, and large initial collapse as risk factors for conservative treatment failure. Our cohort, with a mean T-score of −2.61 and a predominantly elderly, low-BMI female population, falls within this clinically relevant risk stratum. Within this framework, the identification of adjunctive interventions capable of improving pain control and reducing the probability of treatment failure without introducing additional procedural risk is of direct clinical importance. Our data suggest that BioSi-DK supplementation may contribute meaningfully to this objective. The observed kyphoplasty rate of 5.3% in the BioSi-DK group compared with 21.1% in controls, while not reaching statistical significance in this sample, is consistent with an absolute risk reduction in a clinically relevant magnitude (NNT ≈ 6.3) and warrants investigation in larger prospective trials powered for this endpoint. Moreover, the impact of analgesic burden in elderly osteoporotic patients should not be underestimated. In fact, NSAIDs carry well-established risks of gastrointestinal and cardiovascular toxicity and have been associated with impaired fracture healing through inhibition of prostaglandin-mediated signaling pathways [42]. Opioids, while effective for short-term analgesia, are associated with adverse effects including falls, constipation, cognitive impairment, and dependence in the geriatric population [43,44]. The observation that 68.4% of BioSi-DK patients discontinued all analgesics by six months, compared with 36.8% of controls, therefore has implications beyond pain management per se, extending to the reduction in analgesic-related morbidity in a fragile patient population. More broadly, the substantial and frequently persistent pain that accompanies vertebral fractures is itself a major determinant of functional limitation and impaired quality of life, consistent with the disabling impact of spinal pain documented across other populations [45]; achieving durable pain control is therefore a clinically meaningful objective in its own right.

The STIR-sequence MRI was central to the diagnostic and follow-up framework of this study, serving both to confirm fracture acuity at inclusion and to quantify MES evolution at six months. In fact, the Viola et al. [18] study demonstrated, over a ten-year institutional experience, that STIR MRI findings modify treatment plans established on the basis of CT alone in a clinically significant proportion of patients, underscoring the independent diagnostic value of this imaging modality in the OVCF context. In our study, an intermediate MRI evaluation at two months revealed a differential temporal pattern of edema resolution: the BioSi-DK group demonstrated a statistically significant shift toward lower MES grades as early as two months (*p* = 0.003), whereas the control group did not show a significant change at that time point (*p* = 0.06). This earlier radiological response in the supplemented group is consistent with the known osteoblast-stimulating and collagen-promoting effects of bio-activated orthosilicic acid and supports the hypothesis of an accelerated early healing response. By six months, both groups demonstrated substantial MES improvement, with no patient in either group showing edema worsening, a finding consistent with the expected natural history of OVCF repair under conservative management. The absence of a statistically significant between-group difference in MES distribution at six months (Wilcoxon W = 165.5, *p* = 0.620) indicates that edema resolution ultimately followed a comparable trajectory in both groups, with the BioSi-DK effect appearing most pronounced in the earlier phase of the healing process. This finding should be interpreted in context: the primary mechanistic effects of bio-activated silicon, vitamin D3, and vitamin K2 may manifest predominantly at the level of bone matrix quality, mineralization efficiency, collagen network integrity, and pain signaling modulation rather than through the accelerated resolution of the acute inflammatory-edematous response per se. The trajectory of the BME resolution on STIR imaging may thus be an imperfect surrogate for the broader bone quality and pain-related benefits conferred by the supplementation. This interpretation is supported by the work of Zhang et al. [46], who demonstrated that quantitative MRI-based BME parameters correlate with histo-morphometric bone healing indices, including trabecular bone volume and osteoid surface, suggesting that subtle qualitative differences in the healing microenvironment may not be captured by semi-quantitative MES grading alone.

At two-month follow-up, Genant grade distribution was statistically comparable between groups (Wilcoxon W = 178.5, *p* = 0.742; rank-biserial r = 0.05), indicating that the two groups underwent similar early structural evolution and confirming that baseline fracture severity was well balanced. The divergence in vertebral compression outcomes became apparent only at six months: the Genant semi-quantitative grading revealed a noteworthy directional pattern at that time point, with complete Genant grade stability observed in 100% of BioSi-DK patients, compared with vertebral height progression in 16% of controls, including one patient progressing to Grade 3 collapse.

This finding, while statistically underpowered in the current sample, aligns with the mechanistic hypothesis that combined silicon, vitamin D3, and vitamin K2 supplementation may improve the biomechanical resistance of the fracture zone to progressive collapse through enhancement of mineralization quality and trabecular integrity, a biological effect that would be expected to manifest over the six-month follow-up horizon employed in this study. The mechanistic considerations summarized below are drawn from external in vitro, animal, and clinical studies and are presented to provide biological plausibility; the present study did not measure bone turnover markers, osteocalcin carboxylation status, serum 25-hydroxyvitamin D, or any other biomarker; therefore, these pathways were not assessed in our patients. The following synthesis should accordingly be read as context rather than as evidence of mechanism in this cohort, and our conclusions are deliberately framed in clinical rather than mechanistic terms. The inclusion of vitamin D3 in the BioSi-DK formulation is supported by substantial evidence linking vitamin D insufficiency with impaired fracture healing in elderly osteoporotic patients. The active metabolite 1,25-dihydroxyvitamin D [1,25(OH)_2_D] promotes intestinal calcium absorption, enhances renal calcium conservation, and regulates osteoblast–osteoclast coupling through the RANK/RANKL/osteoprotegerin pathway, thereby supporting bone mineralization and skeletal integrity [22]. Vitamin D deficiency, highly prevalent in older populations, has been associated with impaired callus mineralization, reduced biomechanical fracture healing, and an increased risk of secondary fractures [47]. In addition to these systemic endocrine effects, vitamin D also exerts local autocrine actions within bone tissue, directly influencing osteoblast differentiation and bone remodeling [23]. Adequate vitamin D supplementation at the time of fracture may therefore support both local fracture repair and the prevention of systemic bone loss following fracture events [48]. Menaquinone-7 (MK-7), the vitamin K2 isoform included in BioSi-DK, exerts its principal skeletal effect as a cofactor for γ-glutamyl carboxylase, the enzyme responsible for γ-carboxylation of osteocalcin [49]. This modification converts newly synthesized osteocalcin into its calcium-binding form, enabling effective hydroxyapatite integration and bone matrix mineralization [25]. Consequently, elevated levels of uncarboxylated osteocalcin, commonly observed in vitamin K insufficiency, reflect impaired bone mineral quality. Clinical evidence supports this mechanism: a recent meta-analysis confirmed that vitamin K supplementation improves lumbar spine bone mineral density and increases the ratio of carboxylated osteocalcin while reducing uncarboxylated osteocalcin, particularly in older populations [50]. In addition, MK-7 exhibits anti-resorptive effects by increasing osteoprotegerin expression, thereby inhibiting RANKL-mediated osteoclast differentiation through suppression of the NF-κB pathway [51]. Observational studies have further linked higher circulating or dietary vitamin K2 levels with reduced fracture risk [52]. The interaction between vitamins D3 and K2 appears particularly relevant for skeletal repair. Experimental data demonstrate that co-supplementation modulates genes involved in mineralization and angiogenesis while achieving a balanced regulation of osteocalcin expression and γ-carboxylation [53]. Clinical studies have likewise reported greater improvements in bone mineral density with combined vitamin D3 and K2 supplementation compared with either vitamin alone [54,55]. More recently, improved spinal fusion outcomes and bone turnover marker profiles have been observed in elderly osteoporotic patients receiving combined vitamin K2 and D3 therapy, supporting the relevance of this combination in vertebral bone healing contexts [56]. Bio-activated orthosilicic acid (OSA), the silicon component of BioSi-DK, provides an additional biological rationale for enhanced skeletal repair. Silicon is concentrated in actively mineralizing bone and has been shown to stimulate collagen type I synthesis, osteoblast differentiation, and alkaline phosphatase activity in human osteoblast-like cells [57]. Mechanistically, OSA promotes osteogenesis through activation of the PI3K–Akt–mTOR pathway and upregulation of RUNX2 and other osteogenic markers [58]. Importantly, OSA also suppresses osteoclast differentiation by inhibiting RANKL-mediated signaling pathways, suggesting complementary anti-resorptive effects alongside vitamin K2 [26]. Experimental studies have further demonstrated synergistic osteogenic activity when OSA and vitamin K2 are combined, with enhanced expression of RUNX2, ALP, and collagen genes, particularly in senescent mesenchymal stromal cells typical of aging bone [59]. Clinical observations support these mechanistic findings. Epidemiological analyses, including the Framingham Offspring cohort, have reported positive associations between dietary silicon intake and bone mineral density [60], while systematic reviews have similarly identified correlations between silicon intake and improved skeletal outcomes [61]. In a randomized trial, supplementation with choline-stabilized orthosilicic acid significantly improved markers of collagen formation compared with calcium and vitamin D alone [30]. Experimental work has also shown protective vascular and osteogenic effects of OSA in bone injury models, highlighting its potential relevance to fracture repair in compromised microvascular environments [62]. These observations also sit within a wider and growing interest in nutraceutical and plant-derived adjuncts for osteoporosis and fracture healing. For example, a recent narrative review of Cissus quadrangularis has summarized preclinical and clinical evidence for dual anabolic and anti-resorptive actions on bone, illustrating that several complementary agents are being explored as adjuncts to standard osteoporosis care while still awaiting confirmation in adequately powered randomized trials [63]. The present formulation should be viewed within this broader, still-evolving evidence base rather than in isolation.

From a clinical perspective, optimizing conservative management of OVCFs remains essential, as vertebral augmentation procedures, although very effective for short-term pain relief, carry potential risks including cement leakage and adjacent-level fractures [64,65]. In our cohort, vertebral augmentation was required less frequently in patients receiving BioSi-DK supplementation, suggesting a potential role for this strategy in reducing treatment failure during conservative management. Although this difference did not reach statistical significance, the magnitude of effect is clinically relevant and may have implications for reducing the cascade of subsequent OVCFs associated with vertebral augmentation procedures.

### Strengths and Limitations

This study has several methodological strengths that distinguish it from prior descriptive reports in the nutritional supplementation literature. The use of PSM with sex-exact constraint and a stringent caliper achieved excellent covariate balance across all twelve pre-specified baseline variables, substantially mitigating the confounding inherent to non-randomized treatment allocation. The pre-specified IPTW sensitivity analysis, using an independent weighting method, confirmed the robustness and internal consistency of the primary pain outcome across both analytical frameworks. All treatment effect estimates were reported with 95% confidence intervals and standardized effect sizes, enabling quantitative interpretation of findings beyond the binary significance threshold. The inclusion of an intermediate two-month MRI assessment alongside the six-month endpoint provided longitudinal coverage of both the early and late phases of the primary healing period of OVCFs, enabling detection of differential temporal trajectories in MES resolution and vertebral structural stability between groups. The zero adverse event rate associated with BioSi-DK supplementation across the entire study period establishes an acceptable safety profile in this frail, elderly population.

However, these strengths must be weighed against several limitations. First, the retrospective design introduces the inherent risk of unmeasured confounding. PSM and IPTW can only adjust for observed covariates; residual confounding from variables not captured in the institutional record, such as dietary calcium and vitamin D intake, physical activity level, pre-existing vitamin K status, serum 25-hydroxyvitamin D concentrations, frailty status, rehabilitation intensity, socioeconomic factors, and adherence to prescribed anti-osteoporotic pharmacotherapy, cannot be excluded and may have influenced fracture healing trajectories independently of the intervention. In particular, although all patients received vitamin D supplementation as part of standard osteoporosis care, baseline serum 25-hydroxyvitamin D concentrations were not systematically recorded; because vitamin D deficiency can independently impair fracture healing and modulate pain, the absence of baseline vitamin D status represents a notable limitation, and future prospective studies should document it together with vitamin K status and bone turnover markers. A further important limitation is that no formal functional or disability outcome measures (such as the Oswestry Disability Index, the Roland–Morris Disability Questionnaire, mobility scores, or quality-of-life instruments) were available in the institutional records; pain intensity alone may not fully capture clinical recovery, and these patient-reported functional outcomes should be incorporated in future prospective trials. Finally, external validity is constrained by the inclusion criteria: only AO Spine OF1–OF2 fractures managed conservatively were studied, so the present results cannot be generalized to more severe or unstable osteoporotic fractures, to fractures with neurological compromise, or to surgically treated patients. Although MRI follow-up at two months was available for all patients in the matched cohort and enabled the assessment of early radiological changes in marrow edema and vertebral structural stability, clinical outcome data at this intermediate time point, including NRS, analgesic use, and functional status, were available for only a very limited number of patients, precluding any meaningful statistical comparison and therefore not included in the analysis. The two-month assessment was thus restricted to radiological endpoints, while clinical outcomes were only formally evaluated at the six-month follow-up, in accordance with the primary study design. This represents an inherent limitation of the retrospective design, in which intermediate clinical assessments were not systematically collected as part of routine clinical practice, and should be addressed in future prospective trials by incorporating structured clinical evaluations at both two and six months. The sample size is modest. The BioSi-DK group comprised 19 patients, which provides adequate power to detect large effect sizes on the primary pain endpoint, as confirmed by statistically significant NRS results and MES change, but is substantially underpowered for secondary endpoints such as kyphoplasty rate, PGIC responder status, Genant grade progression, for which the confidence intervals are wide, and the non-significant results cannot be interpreted as evidence of absence of effect. Larger multi-center studies are required to achieve definitive conclusions on these endpoints. All patients received anti-osteoporotic pharmacotherapy (including bisphosphonates or teriparatide) in proportions not reported separately per group, and BioSi-DK supplementation is explicitly intended as an adjunct rather than a replacement for these agents. The interaction between BioSi-DK and specific anti-osteoporotic drug classes was not formally evaluated in this study. It is biologically plausible that the magnitude of the BioSi-DK benefit may vary according to concurrent pharmacological treatment, for example, being greater in patients receiving anabolic agents that are already driving osteoblast activity and may therefore provide a more responsive cellular substrate for the silicon-mediated collagen and mineralization effects. Although a validated four-category MES classification was applied, BME quantification relied on semi-quantitative radiologist assessment rather than standardized volumetric or signal intensity ratio measurements. The development and application of quantitative MRI biomarkers of edema resolution and mineral density change would substantially strengthen future investigations and allow the detection of more subtle between-group differences that may escape detection by ordinal semi-quantitative grading. Dual-layer spectral CT, which has demonstrated sensitivity and specificity for BME detection approaching that of STIR MRI, represents a complementary imaging modality that could be incorporated in future prospective follow-up protocols [66]. The follow-up period of six months, while well-aligned with the primary healing phase of OVCFs and the full duration of BioSi-DK administration, may not have been sufficient to capture all clinically relevant longer-term outcomes, including secondary fracture incidence, kyphotic deformity progression beyond six months, bone mineral density changes on DXA, and patient-reported quality of life trajectories at twelve and twenty-four months. These endpoints should be prespecified in future prospective trials. Finally, the international consensus on OVCF conservative management remains absent, and variations in brace design, analgesic regimens, physiotherapy input, and thresholds for surgical escalation across institutions may influence the comparative outcomes against which BioSi-DK supplementation is evaluated [61,62,63].

## 5. Conclusions

Oral supplementation with bio-activated orthosilicic acid combined with vitamins D3 and K2 (BioSi-DK), administered as an adjunct to standard conservative management over six months, was associated with a statistically significant and clinically large reduction in pain at six-month follow-up in patients with acute thoracolumbar OVCFs, alongside a favorable but statistically non-significant trend towards reduced analgesic consumption, lower kyphoplasty rates, greater patient-perceived improvement, and complete vertebral compression grade stability. The primary pain outcome was robust across both PSM and IPTW analytical frameworks. No adverse effects were observed. The biological plausibility of these findings is underpinned by the complementary and synergistic mechanisms through which bio-activated silicon, vitamin D3, and vitamin K2 act across osteoblast differentiation, type I collagen synthesis, osteocalcin γ-carboxylation, hydroxyapatite mineralization, osteoclast suppression, and bone matrix quality, as documented by a substantial and growing body of in vitro, translational, and clinical evidence. Because this was a retrospective, non-randomized study, these findings indicate an association rather than a causal effect and should be regarded as hypothesis-generating. These results are compatible with BioSi-DK being a promising, well-tolerated adjunct to conservative OVCF management, particularly for the primary goal of pain control and the secondary goal of reducing escalation to vertebral augmentation. Validation through prospective, randomized, placebo-controlled trials with larger sample sizes, systematic assessment of nutritional baseline status, longer follow-up, and hard endpoints, including adjacent fracture incidence and bone mineral density, is needed to confirm and extend these initial findings.

## Figures and Tables

**Table 1:** Baseline characteristics of the matched cohort stratified by treatment group.

Variable	Overall (n = 38)	Control (n = 19)	BioSi-DK (n = 19)	*p*-Value	SMD
Age, years (mean ± SD)	71.16 ± 6.48	71.63 ± 7.28	70.68 ± 5.73	0.658	0.145
Female sex, n (%)	4 (10.5)	2 (10.5)	2 (10.5)	1.000	<0.001
BMI, kg/m^2^ (mean ± SD)	22.87 ± 1.36	22.87 ± 1.30	22.87 ± 1.44	1.000	<0.001
T-score (mean ± SD)	−2.61 ± 0.32	−2.59 ± 0.36	−2.62 ± 0.28	0.841	0.066
Baseline NRS pain score (mean ± SD)	8.26 ± 0.95	8.26 ± 0.99	8.26 ± 0.93	1.000	<0.001
Pain duration, weeks (mean ± SD)	5.63 ± 1.91	5.47 ± 2.32	5.79 ± 1.44	0.617	0.164
Minor trauma, n (%)	14 (36.8)	7 (36.8)	7 (36.8)	1.000	<0.001
Diabetes, n (%)	9 (23.7)	5 (26.3)	4 (21.1)	1.000	0.124
COPD, n (%)	5 (13.2)	2 (10.5)	3 (15.8)	1.000	0.156
Cardiovascular disease, n (%)	4 (10.5)	2 (10.5)	2 (10.5)	1.000	<0.001
Hypertension, n (%)	12 (31.6)	6 (31.6)	6 (31.6)	1.000	<0.001
Current smoker, n (%)	17 (44.7)	8 (42.1)	9 (47.4)	1.000	0.106
Bisphosphonate therapy, n (%)	38 (100)	19 (100)	19 (100)	1.000	<0.001
Vitamin D supplementation, n (%)	38 (100)	19 (100)	19 (100)	1.000	<0.001

## Data Availability

The data presented in this study are available on reasonable request from the corresponding author.

## Supplementary Materials

The following supporting information can be downloaded at: https://www.mdpi.com/article/10.3390/jcm15135206/s1, Table S1: Baseline characteristics and covariate balance in the full unmatched cohort (N = 60) and in the propensity-score–matched cohort (n = 38), with standardized mean differences (SMDs) before and after matching.

## Abbreviations

ALP	Alkaline phosphatase
AO Spine	AO Spine (Arbeitsgemeinschaft für Osteosynthesefragen) classification
BMD	Bone mineral density
BME	Bone marrow edema
BMI	Body mass index
BioSi-DK	Bio-activated orthosilicic acid combined with vitamins D3 and K2 (oral dietary supplement)
COPD	Chronic obstructive pulmonary disease
CT	Computed tomography
DXA	Dual-energy X-ray absorptiometry
DGOU	Deutsche Gesellschaft für Orthopädie und Unfallchirurgie (German Society for Orthopaedics and Trauma)
IQR	Interquartile range
IPTW	Inverse probability of treatment weighting
MES	Marrow edema score
MK-7	Menaquinone-7 (vitamin K2 isoform)
MRI	Magnetic resonance imaging
NASS	North American Spine Society
NF-κB	Nuclear factor kappa-light-chain-enhancer of activated B cells
NNT	Number needed to treat
NRS	Numerical rating scale
NSAIDs	Non-steroidal anti-inflammatory drugs
OF1/OF2	Osteoporotic fracture types 1 and 2 (AO Spine classification)
OSA	Orthosilicic acid
OPG	Osteoprotegerin
OVCFs	Osteoporotic vertebral compression fractures
PGIC	Patient global impression of change
PI3K–Akt–mTOR	Phosphoinositide 3-kinase—protein kinase B—mechanistic target of rapamycin signaling pathway
PSM	Propensity score matching
RANK	Receptor activator of nuclear factor kappa-B
RANKL	Receptor activator of nuclear factor kappa-B ligand
ROC	Receiver operating characteristic
RUNX2	Runt-related transcription factor 2
SD	Standard deviation
SMD	Standardized mean difference
STIR	Short tau inversion recovery (MRI sequence)
WHO	World Health Organization
1,25(OH)_2_D	1,25-dihydroxyvitamin D (calcitriol, active metabolite of vitamin D)
