# Supplementary material for: Oral Food Supplement with Bio-Activated Silicium and Vitamins D3 and K2 in the Conservative Management of Osteoporotic Vertebral Compression Fractures
Source: J Clin Med. 2026 Jul 3;15(13):5206. doi: 10.3390/jcm15135206 (PMC13363488; doi:10.3390/jcm15135206)
Supplement: Supplementary file 1 [file jcm-15-05206-s001.zip › jcm-4382155-supplementary.pdf]

Supplementary Material

Supplementary Table S1. Baseline characteristics and covariate balance in the full unmatched cohort (N = 60) and in the propensity-score–matched cohort (n = 38), with standardized mean differences (SMDs) before and after matching.

	**Full unmatched cohort (N = 60)**			**PS-matched cohort (n = 38)**		
**Characteristic**	**Control (n = 41)**	**BioSi-DK (n = 19)**	**SMD**	**Control (n = 19)**	**BioSi-DK (n = 19)**	**SMD**
Age, years - mean (SD)	71.88 (7.46)	70.68 (5.73)	0.179	71.63 (7.28)	70.68 (5.73)	0.145
Male sex - n (%)	5 (12.2)	2 (10.5)	0.053	2 (10.5)	2 (10.5)	<0.001
BMI, kg/m² - mean (SD)	22.65 (1.61)	22.87 (1.44)	0.145	22.87 (1.30)	22.87 (1.44)	<0.001
Lumbar T-score - mean (SD)	-2.65 (0.37)	-2.62 (0.28)	0.093	-2.59 (0.36)	-2.62 (0.28)	0.066
Baseline NRS pain - mean (SD)	8.22 (0.99)	8.26 (0.93)	0.045	8.26 (0.99)	8.26 (0.93)	<0.001
Pain duration, weeks - mean (SD)	4.88 (2.00)	5.79 (1.44)	**0.523**	5.47 (2.32)	5.79 (1.44)	0.164
Minor trauma - n (%)	21 (51.2)	7 (36.8)	**0.293**	7 (36.8)	7 (36.8)	<0.001
Diabetes mellitus - n (%)	12 (29.3)	4 (21.1)	0.190	5 (26.3)	4 (21.1)	0.124
COPD - n (%)	4 (9.8)	3 (15.8)	0.181	2 (10.5)	3 (15.8)	0.156
Cardiovascular disease - n (%)	9 (22.0)	2 (10.5)	**0.314**	2 (10.5)	2 (10.5)	<0.001
Hypertension - n (%)	13 (31.7)	6 (31.6)	0.003	6 (31.6)	6 (31.6)	<0.001
Current smoker - n (%)	12 (29.3)	9 (47.4)	**0.379**	8 (42.1)	9 (47.4)	0.106
Mean SMD (max)			**0.222 (0.523)**			**0.063 (0.164)**

Data are mean (standard deviation) for continuous variables and n (%) for categorical variables. The standardized mean difference (SMD) quantifies between-group imbalance independently of sample size; an SMD < 0.10 indicates good balance and an SMD < 0.20 acceptable balance. SMD values ≥ 0.20 in the unmatched cohort are shown in bold. Before matching, the mean SMD across the twelve covariates was 0.222 (maximum 0.523, for pain duration); after matching the mean SMD fell to 0.063 (maximum 0.164) and no covariate exceeded the 0.20 threshold.

Propensity-score method. Propensity scores were estimated by multivariable logistic regression including the twelve pre-specified covariates listed above (age, sex, BMI, lumbar T-score, baseline NRS, pain duration, minor-trauma mechanism, diabetes mellitus, COPD, cardiovascular disease, hypertension, and smoking status). Matching was 1:1 greedy nearest-neighbour on the logit of the propensity score, with a caliper of 0.3 SD of the logit-propensity score (caliper width = 0.3192). A sex-exact constraint was imposed as the primary matching criterion, with a hierarchical fallback (sex-exact within caliper → sex-exact without caliper → unconstrained nearest-neighbour) that retained all 19 BioSi-DK patients. The propensity-score distribution showed adequate common support (PS range 0.033-0.829).

Abbreviations: BioSi-DK, bio-activated orthosilicic acid plus vitamins D3 and K2; BMI, body mass index; COPD, chronic obstructive pulmonary disease; NRS, numeric rating scale; PS, propensity score; SMD, standardized mean difference.

Note. Sex is reported as male n (%), consistent with the analysis dataset (full cohort: 7/60 male, 88.3% female). One cerebrovascular event (stroke) was recorded in the BioSi-DK group of the unmatched cohort but was not among the twelve pre-specified matching covariates and is therefore not tabulated. Group sizes differ from a 1:1 ratio in the unmatched cohort (41 control vs 19 BioSi-DK) because allocation was non-randomized.
